# Evaluating the reliability of the athlete sleep behavior questionnaire (ASBQ): a meta-analysis of Cronbach’s alpha and intraclass correlation coefficient

**DOI:** 10.1186/s13102-023-00787-0

**Published:** 2024-01-02

**Authors:** Khaled Trabelsi, Zahra Saif, Matthew W Driller, Michael V. Vitiello, Haitham Jahrami

**Affiliations:** 1 High Institute of Sport and Physical Education of Sfax, University of Sfax, 3000 Sfax, Tunisia; 2 Research Laboratory: Education, Motricity, Sport and Health, EM2S, LR19JS01, University of Sfax, 3000 Sfax, Tunisia; 3 Government Hospitals, Manama, Bahrain; 4 SIESTA Research Group, School of Allied Health, Human Services and Sport, La Trobe University, 3086 Melbourne, VIC Australia; 5 Sport, Performance, and Nutrition Research Group, School of Allied Health, Human Services and Sport, La Trobe University, 3086 Melbourne, VIC Australia; 6 Department of Psychiatry & Behavioral Sciences, University of Washington, Seattle, USA; 7 Department of Psychiatry, College of Medicine and Medical Sciences, Arabian Gulf University, Manama, Bahrain

**Keywords:** ASBQ, Circadian rhythm, Sleep, Reliability generalization, Meta-analysis

## Abstract

**Background:**

The Athlete Sleep Behavior Questionnaire (ASBQ) was designed to identify maladaptive sleep practices among athletes. The aim of this meta-analysis was to evaluate the internal consistency and the test-retest reliability coefficients of the ASBQ.

**Methods:**

A systematic search across 10 databases from inception of the ASBQ to August 2023 was performed. Publications that reported estimates of internal consistency and/or test-retest reliability of the ASBQ were included. A random-effects model was employed to estimate the overall reliability measures of the ASBQ.

**Results:**

Meta-analytic results demonstrated a good level of internal consistency within the ASBQ, evidenced by a Cronbach’s alpha of 0.73 (95% CI: 0.63 to 0.80). This suggests a modest correlation among the questionnaire items, supporting its reliability as an effective measure of sleep behavior. In terms of test-retest reliability, our meta-analysis revealed a very good degree of consistency (ICC = 0.88; 95% CI: 0.87 to 0.89), suggesting that the ASBQ can serve as an instrument for monitoring and evaluating changes in athletes’ sleep behavior over time. No evidence of publication bias was identified.

**Conclusion:**

While the ASBQ demonstrates a moderate level of internal consistency, its test-retest reliability suggests that it can serve as an instrument for longitudinal assessments of athletes’ sleep behavior. Future studies focusing on refining the ASBQ to optimize its internal consistency and validate its applicability across diverse athletic populations are warranted.

## Introduction

Sleep plays a vital role in the well-being and performance of athletes [1]. Adequate and high-quality sleep is essential for various physiological (e.g., muscle recovery, hormone regulation, metabolism optimization, immune system regulation) and psychological (e.g., memory consolidation, attention, decision-making, mood stability) processes that directly affect athletic performance [2, 3]. However, a significant number of athletes often experience sub-optimal sleep quantity and/or quality due to compromised general sleep behaviors [2, 3]. These compromised behaviors often arise from several physiological factors, such as the increase of core temperature post-exercise, increased muscle tension, post-training or competition fatigue or discomfort, and stress before important competitions [4–9]. Additionally, behavioral factors such as excessive and/or untimely caffeine consumption, along with environmental disruptions such as disturbances from light and noise and frequent transmeridian travel, also contribute to these challenges [4–9]. Such circumstances can have detrimental effects on athletes’ performance and overall health, increasing their susceptibility to injuries and infections [4, 10]. As a result, it is essential to (i) regularly assess and monitor athletes’ sleep using both objective (e.g., actigraphy) and subjective (e.g., questionnaires, scales) tools, and (ii) identify modifiable factors potentially contributing to sub-optimal sleep in order to offer targeted interventions for improving athletes’ sleep behaviors [4–6].

Although various tools for sleep assessment are available, the Athlete Sleep Behavior Questionnaire (ASBQ) [11, 12] is the only existing self-report instrument specifically designed to evaluate behavioral factors that may influence sleep quantity/and/or quality in athletes [11, 12]. The ASBQ consists of 18 items that cover a wide range of sleep-related aspects, such as sleep routines, quality, latency, disturbances, and the use of sleep aids [11, 12]. Athletes are asked to rate their sleep behavior and experiences using established response scales [11, 12]. The ASBQ is a standardized tool that can help researchers and sports medicine professionals learn important details about athletes’ sleep habits and identify any issues or areas that need to be improved.

Given the significance of the ASBQ as a measurement tool in sports medicine research, it is important to evaluate its psychometric properties, with a particular focus on reliability. Reliability refers to the consistency and stability of the questionnaire’s measurements over time or across different raters. In the case of the ASBQ. Reliability can be assessed using measures such as Cronbach’s alpha and the intraclass correlation coefficient (ICC) [13]. Cronbach’s alpha is commonly used to evaluate the internal consistency and reliability of multi-item scales, providing an estimate of how well the items in the questionnaire measure the same underlying construct [14]. A higher Cronbach’s alpha indicates stronger internal consistency, indicating that the scale’s items are highly correlated and collectively measure the targeted construct reliably [14]. The ICC is a measure for assessing agreement between different raters or repeated measurements, such as test-retest reliability [15]. It quantifies the proportion of the total variance in the measurements that can be attributed to differences between subjects or items of interest, relative to the total variance [15]. A higher ICC value indicates stronger agreement and consistency in the measurements, indicating that the ASBQ yields stable results over time or across different raters [15].

The ASBQ was initially developed in English in 2018 [12], and since then, translations into Turkish [16], Portuguese [17], French [18], and Arabic [19] have been completed. Translation into other languages is anticipated and thus having early pooled results of the scale’s reliability is important for the ASBQ’s continued adoption and global applicability. Conducting a meta-analysis of the reliability estimates of the ASBQ is essential to synthesize existing evidence and provide a comprehensive evaluation of its reliability across multiple studies. By pooling data from various studies, a meta-analysis can offer a more robust and precise estimation of the ASBQ’s psychometric properties and provide valuable insights to researchers and practitioners regarding the overall quality and usefulness of the instrument in assessing sleep behavior among athletes. To the best of our knowledge, this is the first review to synthesize the evidence base and reliability metrics of the ASBQ.

## Methods

The Preferred Reporting Items for Systematic Reviews and Meta-Analyses (PRISMA 2020) statement [20], and the REliability GEneralization Meta-Analysis statement (REGEMA) were used to guide this meta-analysis [21]. The PICO (i.e., P = Population, I = Intervention, C = Comparison, and O = Outcome) review question to facilitate: What is the overall reliability of the ASBQ? (I) internal consistency and intraclass reliability (O) in Athletes and non- Athletes (P) No comparison were applicable (C).

### Literature search

To identify relevant studies reporting reliability estimates of internal consistency and test-retest reliability of the Athlete Sleep Behavior Questionnaire (ASBQ), a systematic search was conducted between January 2018 (i.e., inception of ASBQ) and August 2023. Ten databases were searched, including CINAHL (Cumulative Index to Nursing and Allied Health Literature), Cochrane Library, Embase, ERIC (Education Resources Information Center), PEDro (Physiotherapy Evidence Database), PsycINFO, PubMed, Scopus, SPORTDiscus, and Web of Science. The search terms “Athlete Sleep Behavior Questionnaire” OR “ASBQ” AND “psychometric properties” OR “reliability” OR “validity” were utilized. Manual searches of reference lists from relevant articles, reviews, and preprints were performed to identify any additional studies. Two authors (HJ, ZS) separately performed literature search and subsequent screening. The two authors are experts in sleep medicine and systematic reviews, with a doctoral degree in their respective fields.

### Selection criteria

For inclusion in the meta-analysis, studies had to meet certain criteria. Firstly, they needed to report internal consistency estimates of the ASBQ using Cronbach’s alpha (α) or equivalent e.g. McDonald’s Omega, average inter-item correlation, split-half reliability, Guttman’s Lambda, and/or test-retest reliability estimates using the intraclass correlation coefficient (ICC) or equivalent e.g. Cohen’s Kappa, Fleiss’ Kappa, Krippendorff’s Alpha. Secondly, the studies had to be published in English language. Thirdly, the ASBQ or its translated versions had to be utilized as a self-report instrument for validation purposes as the primary instrument. Lastly, the studies needed to provide sufficient data for the calculation of effect sizes. Studies employing non-standard versions of the ASBQ (e.g., short form or partial) and studies with no or insufficient data to determine reliability coefficients were excluded.

The citation management system utilized was EndNote 21 for Windows (*.RIS). EndNote was also used to delete duplicates and automate deletion of false positive e.g. studies with other ASBQs such as An Arabic Sedentary Behaviors Questionnaire or Assessment of Suicidal Behaviors Questionnaire.

### Data extraction and study characteristics

Relevant data were independently extracted by two authors (ZS and HJ), and any discrepancies were resolved through discussion with third author (KT). The extracted information included study characteristics such as sample size, participant age, and more. Additionally, reliability estimates and potential factors influencing reliability, such as study design, setting, and administration method, were recorded.

### Quality evaluation of the studies

To assess the quality of the included studies, a modified form of the COSMIN [22] (COnsensus-based Standards for the selection of health status Measurement INstruments) critical appraisal tool was used. The COSMIN checklist consists of four domains including reliability, validity, responsiveness, and interpretability [22]. To assess the methodological rigor of included studies, two independent reviewers completed quality appraisal for each study. These two reviewers were the same researchers who performed study screening and data extraction (i.e., ZS and HJ). Interrater agreement was 100%, indicating strong consistency in application of the tool across raters.

### Data analysis

A random-effects meta-analysis using restricted maximum likelihood estimation was conducted to pool Cronbach’s alpha/ICC values across studies [23]. Cronbach’s alpha and the ICC are correlation coefficients that range from 0 to 1, measuring the internal consistency and reliability of scales or ratings [24]. By using these values as outcome metrics in a meta-analysis, we were able to synthesize this reliability evidence across multiple studies. A random-effects model, rather than a fixed effect model, was selected to allow for expected heterogeneity in effects between studies [25]. Untransformed alpha and ICC estimates and their associated inverse variance weights from each study were inputted into the model [26]. This enabled smaller studies with less precision to be weighted appropriately rather than distorting the pooled estimate [25]. By meta-analyzing reliability coefficients in this fashion, we obtained a pooled point estimate and 95% confidence interval to quantify the overall reliability of the construct or ratings assessed by the included scales or raters. Heterogeneity among studies was assessed using statistical measures such as the Q statistic, tau, tau2, and the I² index [27]. These metrics aid in evaluating the degree of variation among the included studies [27]. Studentized residuals and Cook’s distances were used to explore the potential presence of outliers and influential studies within our models [28]. To identify potential outliers, we employ a threshold derived from the 100 × (1–0.05/(2 X k))th percentile of a standard normal distribution, considering a Bonferroni correction with a two-sided alpha of 0.05 for the k studies included in the meta-analysis [28]. Any study with a studentized residual surpassing this threshold was deemed a potential outlier [28]. For Cook’s distances, we set the criterion for influential studies as those with a value exceeding the median plus six times the interquartile range of the Cook’s distances [28]. Such studies would have significant impact within the model [28].

To assess any potential funnel plot asymmetry, we employed both the rank correlation test and the Egger’s regression test [29]. The standard error of the observed outcomes served as a predictor in these tests, allowing us to examine the presence of any asymmetry within the funnel plot [29]. Contoured funnel plots were constructed to visually assess the potential for publication bias in the meta-analysis [30]. These enhanced plots include superimposed contour lines indicating statistical significance levels, allowing gaps in data points to be scrutinized as possible missing studies [30]. Examining the symmetry and shape characteristics with inclusion of significance contours augments funnel plots’ utility for detecting possible publication bias based on visual inspection. If no reporting biases are present, studies of variable precision should appear evenly dispersed across statistical significance bands without substantial white spaces [30]. Any skew or asymmetry triggered by contours highlighting sparse areas of nonsignificant data calls study inclusion patterns into question [31].

Furthermore, the consistency of effect sizes and possibility of citation bias across the included studies will be visually and statistically assessed using a DOI plot and Luis Furuya-Kanamori (LFK) index [32]. The DOI plot graphs the distribution of study effect estimates along with the precision of those estimates [32]. Examining the spread of data points relative to the null line allows appraisal of heterogeneity. The LFK index provides a quantitative measure of bias by comparing the observed versus expected number of statistically significant studies based on statistical power and thresholds [32]. An LFK index near 0 suggests low risk of citation bias. Values greater than ± 1.96 indicate excess significance bias either towards significant or nonsignificant findings, respectively [33]. Cross-analyzing the DOI plot and LFK index will enable robust evaluation of the credibility of statistically significant meta-analysis results and determine if trim-and-fill procedures are required to adjust for potential asymmetry due to citation bias [32, 33].

All analyses were performed using R for statistical computing version 4.3.0, which was released on 2023-04-21. The package “meta” [26] was used, and statistical significance level was set at p < 0.05 for all analyses.

## Results

**Fig. 1:**
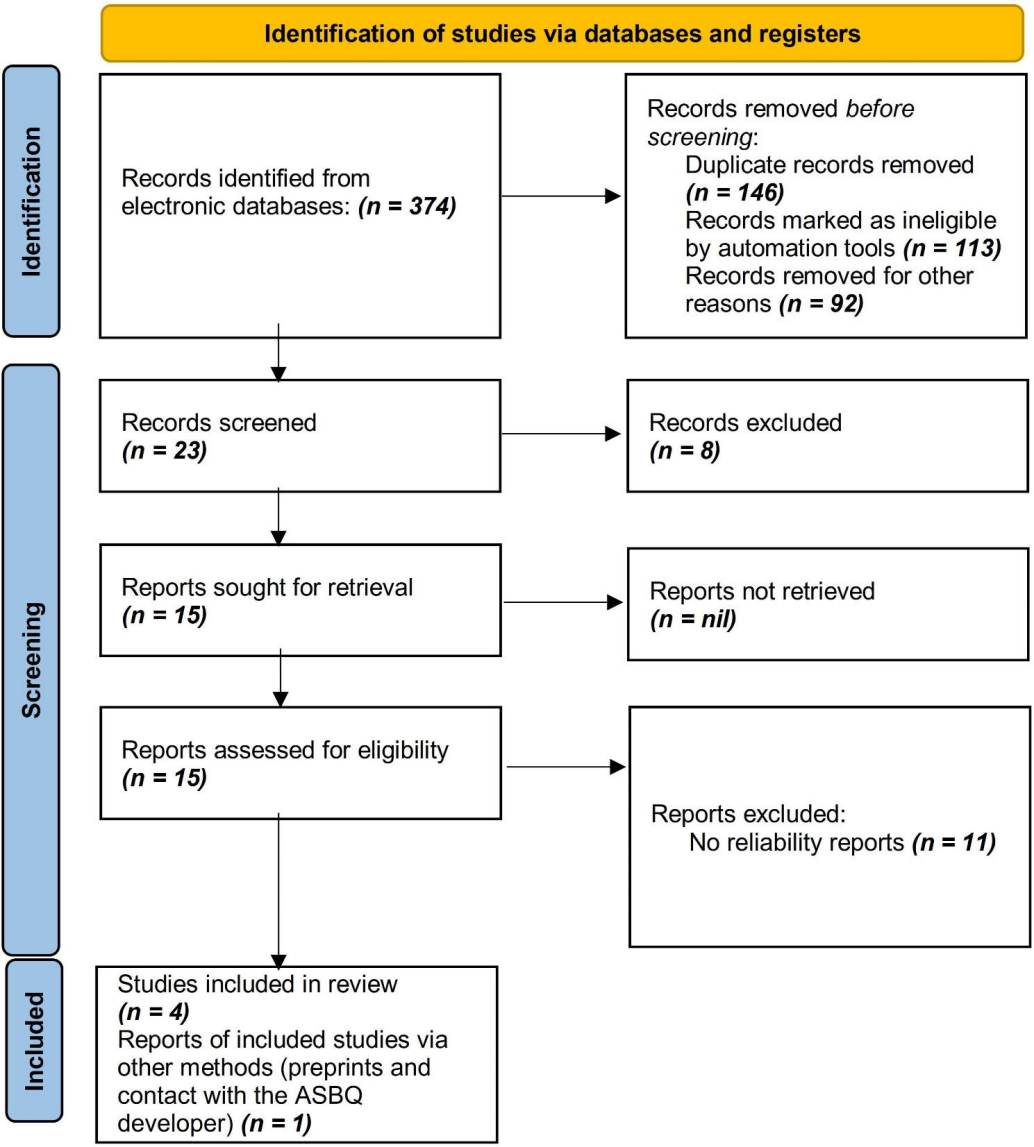
PRISMA flowchart of the study selection

We searched electronic databases and initially identified 374 total records. Before formal screening, we removed duplicates (146) and records marked as clearly ineligible by automation tools (113) or for other reasons (92). The primary reason for exclusion at this stage was disguised ASBQ studies that used research tools with same acronym but different name e.g., Arabic Sedentary Behaviors Questionnaire or Assessment of Suicidal Behaviors Questionnaire. This left 23 records for title and abstract screening. Eight of those did not meet inclusion criteria typically because no reliability measure was reported, or the study involved the wrong patient population. A total of 15 records underwent full text review with a further 11 articles excluded, mainly because they did not report quantitative reliability metrics like a Cronbach’s alpha or intraclass correlation coefficient. This left five studies that were included for data extraction and quantitative synthesis. Figure 1 shows PRISMA flowchart of the study selection. A total of five studies (K = 5) [12, 16–19], comprising N = 1544 participants, were included in this review. In all of the included studies Cronbach’s alpha and ICC were the only methods respectively used to assess internal consistency and reliability of measurements taken on the same subjects over time. Table 1 provides a comprehensive overview of the studies included in the review. Sample sizes range from 52 to 564 participants, with an average of 309 participants. Study participants were aged between 20 and 27 years, with an average age of 23.2. There was a slightly higher proportion of males in the overall sample (57.2%). The Cronbach’s α values ranged from 0.62 to 0.84, indicating variability in internal consistency levels within the ASBQ. Additionally, these Cronbach’s α values suggest a moderate to high level of internal consistency in the ASBQ. The ICC values range from 0.78 to 0.88, indicating consistent to strong agreement between repeated measurements over time. In terms of COSMIN, assessing methodological quality, four studies were rated as “Moderate”, while one study was rated as “Low”.

**Table 1:** Characteristics of included studies

SN	Study	Country/Countries	Study design/Setting*	Mode of Administration	Language	^Mean^ Age	^% Male^ Sex Male: Female	Sample (n)	α	ICC	COSMIN
1	Driller 2018	Australia, Canada, England, India, Malaysia, New Zealand, Portugal, Sweden, and USA	Cross-sectional/Athletes and non-Athletes	Online	English	24 years	50% 282: 282	564	0.63	0.88	Moderate
2	Anketi 2019	Turkey	Cross-sectional/Athletes and non-Athletes	Online	Turkish	21 years	49% 96: 84	180	0.62	0.85	Moderate
3	Facundo 2021	Brazil	Cross-sectional/Athletes	Online	Portuguese	27 years	79% 41: 11	52	0.78	0.86	Low
4	Baize 2023	France	Cross-sectional/Athletes	Online	French	20 years	57% 164: 126	290	0.84	0.88	Moderate
5	Trabelsi 2023*	Bahrain, Jordan, Saudi Arabia, and Tunisia	Cross-sectional/Athletes and non-Athletes	Online	Arabic	24 years	52% 236: 220	458	0.72	0.88	Moderate

**Notes**: *Athletes were recruited from Elite and National Teams Settings. Non-Athletes were recruited from the general population community settings. **Data available as preprint.

SN = Serial number.

α = Cronbach’s alpha for internal consistency.

ICC = intraclass correlation coefficient for test-retest reliability.

COSMIN = COnsensus-based Standards for the selection of health Measurement Instruments.

### Meta-analyses of statistical reliability measures

Table 2 presents the results of meta-analyses of two statistical reliability measures, namely Cronbach’s α and the ICC.

**Table 2:** Meta-analysis of Cronbach alpha and ICC of the ASBQ

Study	Coefficient	LCI 95%	HCI 95%	I^2^	Cochran’s Q	Chi^2^, p	tau^2^	Plot
Cronbach alpha	0.73	0.62	0.80	92.00	50.00	0.00	0.04	Figure 2
ICC	0.88	0.86	0.89	0.00	2.53	0.64	0.00	Figure 3

**Notes**: Random-effect meta-analysis using raw coefficients.

LCI = Lower-limit confidence interval; HCI = Higher-limit confidence interval.

### Cronbach’s α analysis (internal consistency)

**Fig. 2:**
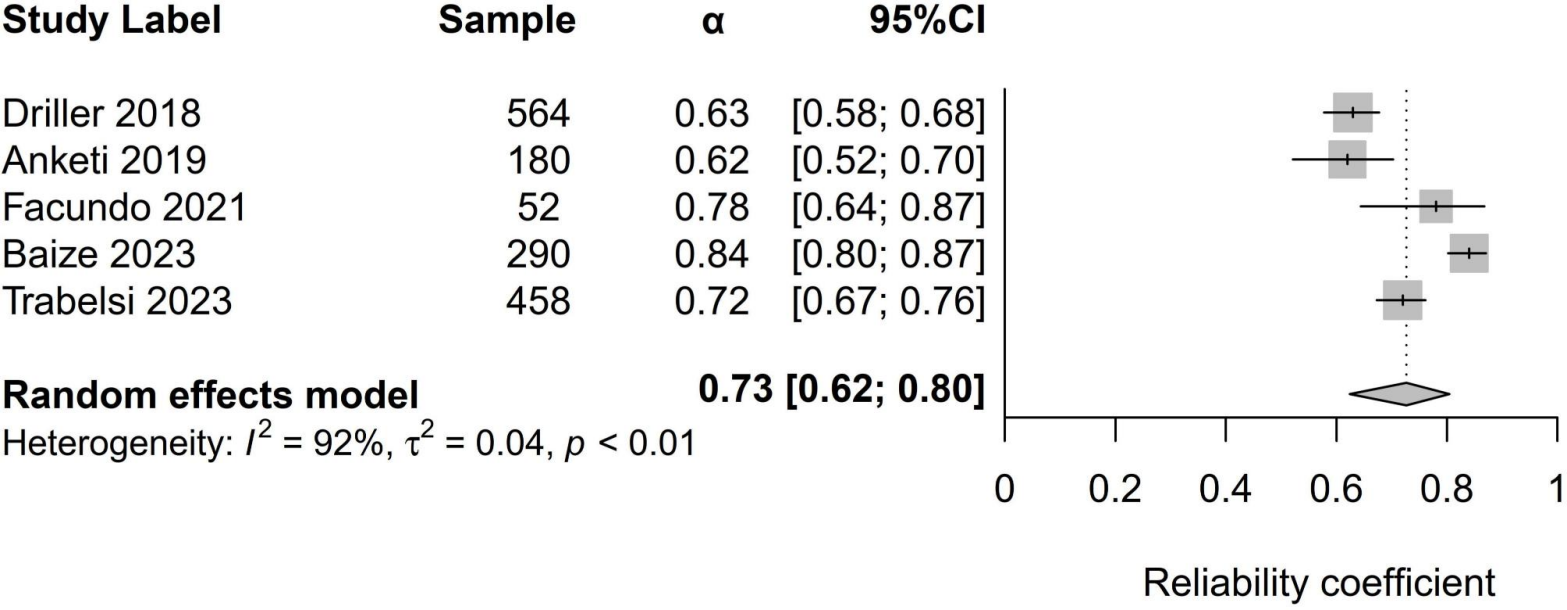
Forest plot of Cronbach alpha the ASBQ

The analysis incorporated Cronbach’s α from five studies, including 1544 participants. Utilizing the random-effects model, an estimated average correlation coefficient of 0.72 (95% CI: 0.63 to 0.80) was computed, Fig. 2. This observation indicates that the average result significantly deviates from zero (z = 16.56, p < 0.001). After performing the Q-test, we identified heterogeneity in the true outcomes (Q(4) = 50, p < 0.0001, tau² = 0.01, I² = 91.66%). The 95% prediction interval for the true outcomes was 0.52 to 0.92. While some degree of heterogeneity is present, it is evident that the true outcomes of the studies generally correspond with the estimated average outcome. Our estimate falls upon the studentized residuals, revealing no values exceeding the threshold of ± 2.58. Therefore, we find no indication of outliers within the framework of this model. Cook’s distances also confirm that none of the studies exerted influence over our analysis. Employing the method of removing one study at a time demonstrates that no individual study affected the outcome by more than 2%. Examination of funnel plot, Fig. 4 and DOI plot, Fig. 5 indicated the absence of publication bias.

**Fig. 3:**
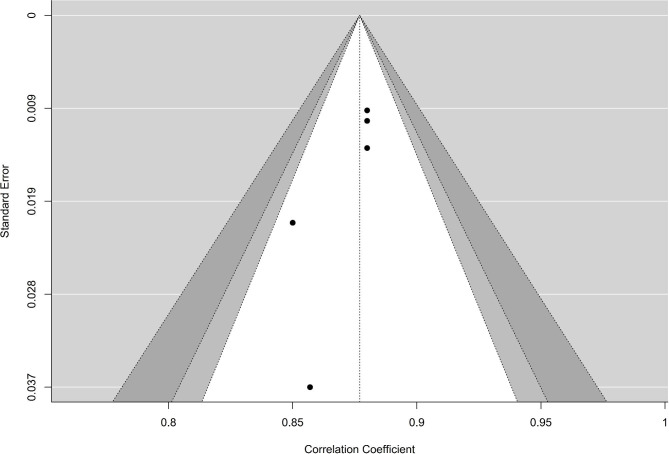
Forest plot of ICC of the ASBQ

**Fig. 4:**
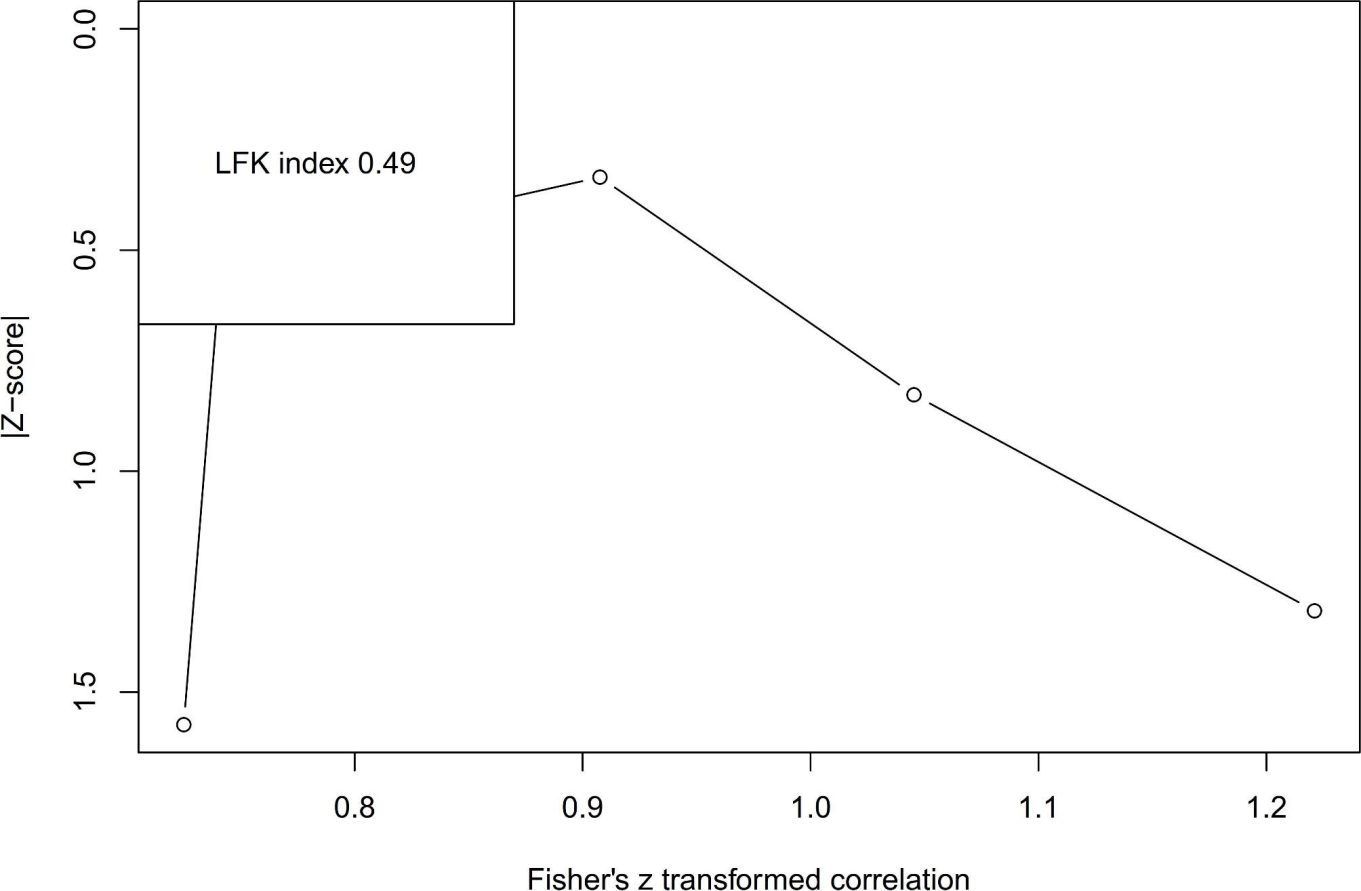
Funnel plot of Cronbach alpha the ASBQ

**Fig. 5:**
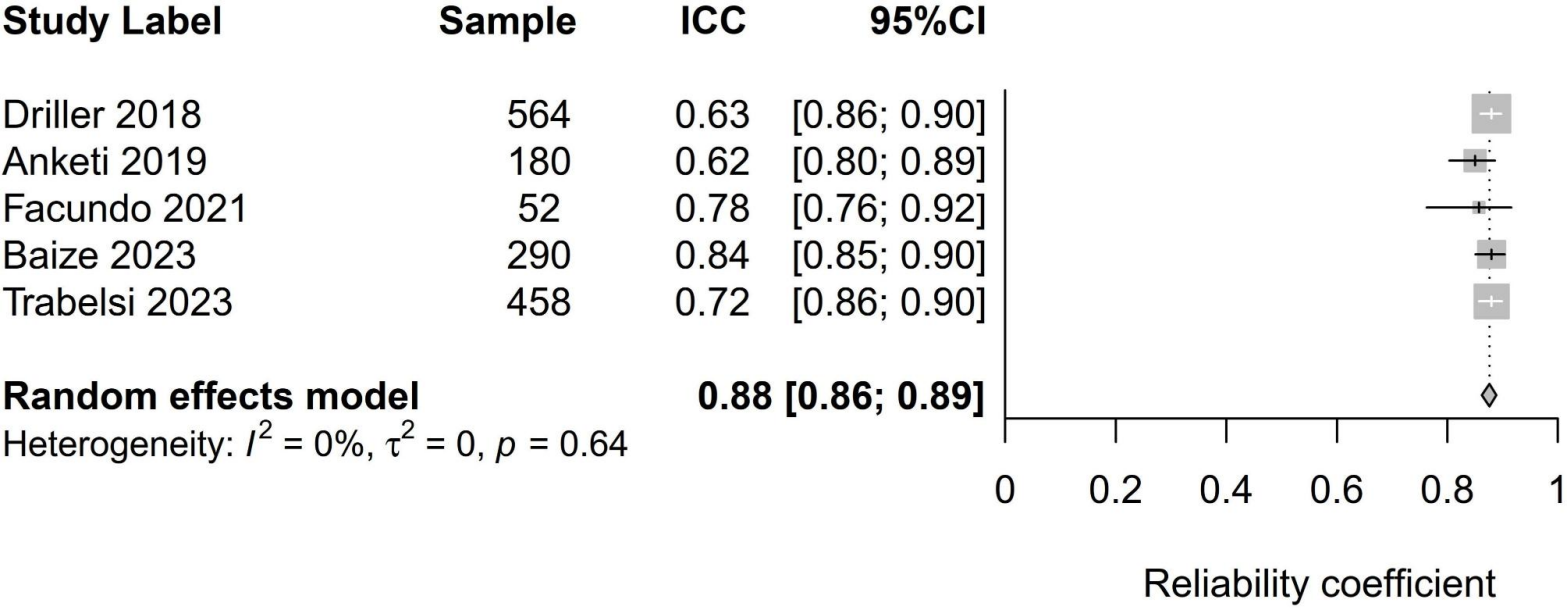
DOI plot of Cronbach alpha the ASBQ

### ICC analysis

**Fig. 6:**
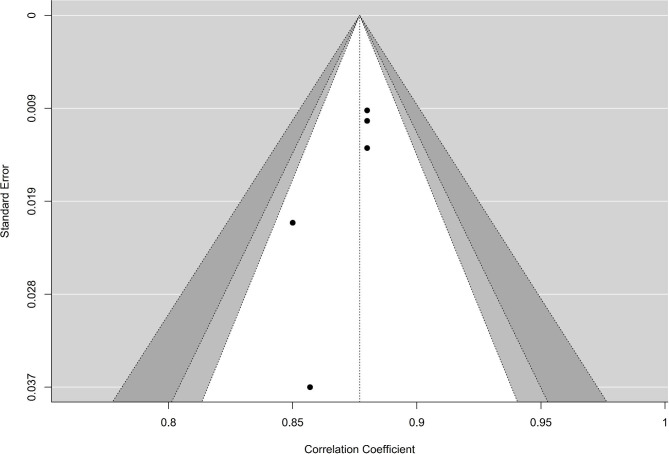
Funnel plot of ICC of the ASBQ

**Fig. 7:**
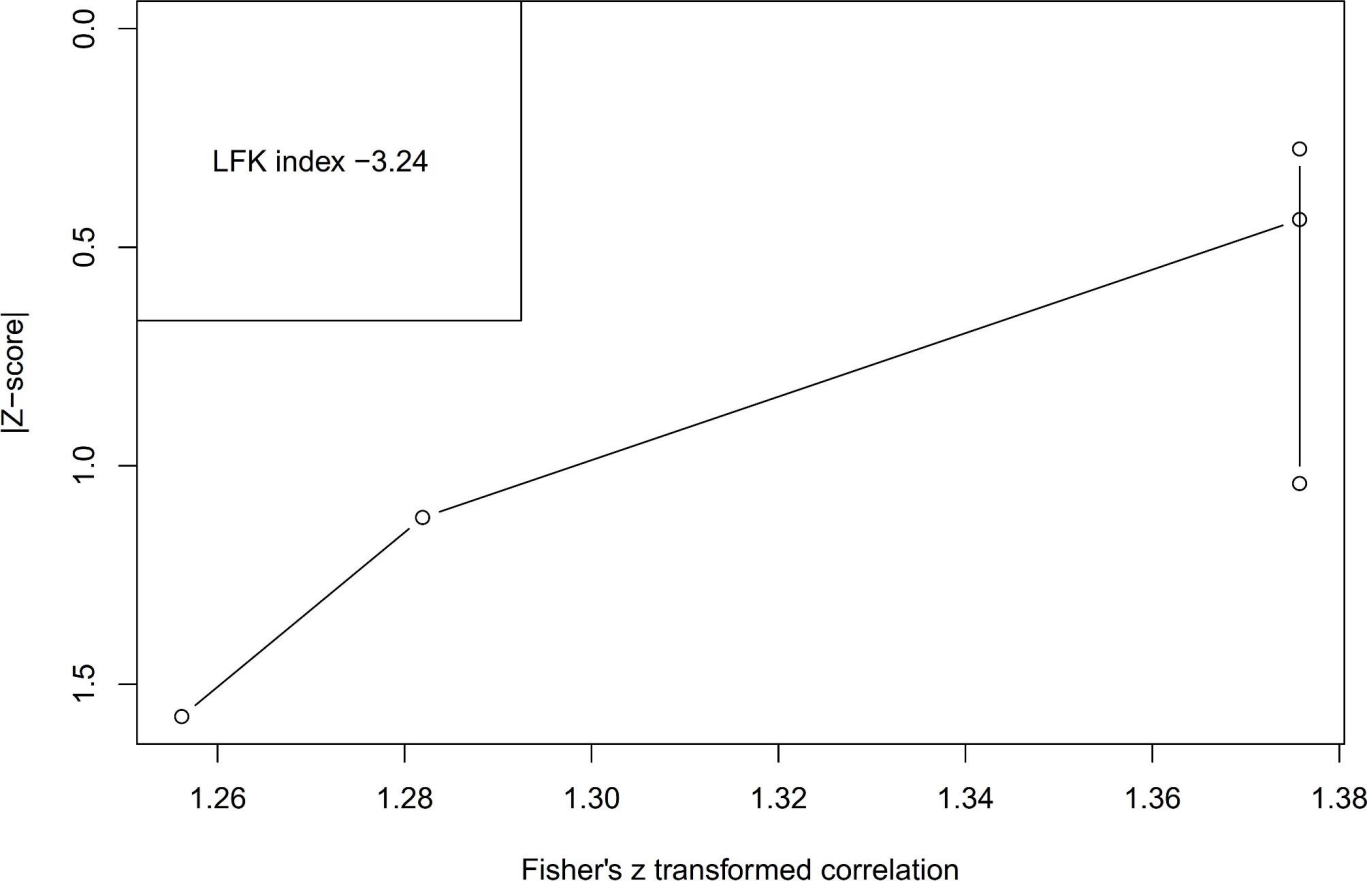
DOI plot of ICC of the ASBQ

A total of 1544 participants from five studies were included in the analysis. Under the framework of the random-effects model, our analysis yielded an estimated average correlation coefficient of 0.88 (95% CI: 0.87 to 0.89), Fig. 3. This finding demonstrates that the average outcome significantly differs from zero (z = 148.75, p < 0.001). Upon applying the Q-test, we found no evidence indicating heterogeneity among the true outcomes (Q(4) = 2.22, p = 0.64, tau² = 0.00, I² = 0.00%). Exploring the studentized residuals, we identified no values exceeding the threshold of ± 2.58. Thus, no indication of outliers was found within this model’s context. Cook’s distances confirmed that none of the studies exerted an excessive influence on our analysis. Employing a one-study-at-a-time removal strategy revealed that no single study had a significant impact on the overall outcome by more than 2%. Examination of funnel plot, Fig. 6 and DOI plot, Fig. 7 indicated the absence of publication bias.

## Discussion

This reliability generalization meta-analysis assessed the consistency and test-retest reliability of the ASBQ. The ASBQ was found to have a moderate level of internal consistency and a high degree test-retest reliability.

The reliability generalization estimates of Cronbachʼs alpha for the ASBQ (α = 0.73) is above the widely accepted cut-off point of 0.70, which indicates an acceptable to good level of internal consistency. It should be acknowledged that Cronbachʼs alpha of the initial English version of the ASBQ was questionable (α = 0.63) [12], whereas the alpha values for the translated versions were either acceptable or good. Driller et al. (2018) noted that Cronbach’s alpha could be influenced by the number of items in the scale [12], with a lower alpha value the result of the relatively small number of items in the ASBQ, rather than indicating low internal consistency per se. Furthermore, the diverse range of items within the ASBQ could reflect the multifaceted nature of sleep behavior in athletes, which is difficult to capture within a single measurement dimension. Consequently, although the value falls below the recommended threshold, it may not necessarily imply that the ASBQ lacks reliability as an assessment tool. A key limitation is that alpha assumes all scale items are equally reliable, however this assumption is often violated in practice. It also assumes a unidimensional factor structure, while many constructs are multidimensional [34]. Alpha is therefore sensitive to the number of items in a scale, where adding redundant or unrelated items can artificially inflate scores [34]. Alpha also has a tendency toward underestimation in short scales with few items [35]. Sole reliance on alpha is not ideal given contemporary perspectives on its weaknesses which include sensitivity to scale length, dimensional inaccuracy with multidimensional scales, and item intercorrelation inequalities [35]. Some researchers argue alpha should be avoided in favor of more modern methods such as McDonald’s omega with its less restrictive assumptions. However, as alpha remains very familiar and accessible, supplementing rather than fully supplanting it may best address its limitations.

Unfortunately, alternative measures of internal consistency, such as McDonald’s Omega, were not explored by Driller et al. (2018) [12] or subsequent translations. McDonald’s Omega is particularly valuable because it considers the interrelationship between items and offers a more precise representation of the instrument’s reliability across diverse dimensions and variations in item responses. This measure provides a valuable complement to Cronbach’s alpha, especially when dealing with complex constructs that might demonstrate multidimensionality. Future validation studies should explore alternative methods (e.g., McDonald’s Omega) to assess the internal consistency of the ASBQ, which is essential for achieving a comprehensive understanding and interpretation of its psychometric properties, thereby enhancing the instrument’s reliability in research and practice.

In the Arabic version of the ASBQ, results revealed an acceptable level of internal consistency (Cronbach’s α = 0.723; McDonald’s ω = 0.725) [19]. However, prior to initiating the translation process, the authors modified item 2 “I use stimulants when I train/compete (e.g., caffeine)” in the original ASBQ version to read as “I use stimulants when I train/compete (e.g., caffeine) after midday”. This adjustment was made considering that many athletes consume caffeine in the morning without it necessarily having an impact on their sleep. Additionally, the results of the item response theory indicated that item 4 of the ASBQ appears to present the highest level of difficulty for respondents. Therefore, the authors recommended that additional refinements to the Arabic version of the ASBQ are warranted to enhance its psychometric properties [19]. Item ASBQ 4 was initially “I consume alcohol (or other stimulants e.g., caffeine) within 4 hours of going to bed”. In Arab Muslim culture, alcohol (and other recreational substances/ psychoactive) are considered taboo, and discussing their consumption can be challenging and uncomfortable. This may explain the reason for this specific item difficulty by Arab athletes.

The high ICC value of 0.88 indicates a strong test-retest reliability. This suggests that the ASBQ provides consistent result over time, which is crucial for a tool aiming to measure a construct that is expected to be stable over time, such as sleep behavior. The ICCs of all the included studies were high > 0.88. This consistency is especially important for the ASBQ, which is designed to evaluate a construct (i.e., sleep behavior) that is anticipated to remain relatively stable over time. This high test-retest reliability highlights the instrument’s capacity to consistently capture and measure the intended construct, reinforcing its applicability for research and practice. Moreover, the ASBQ may be a useful tool for targeting specific hygiene education strategies for individual athletes. This may be achieved by focusing on strategies that target items with low scores in the questionnaire (indicative of poor sleep behaviors). For example, Driller et al. (2019) reported that personalized sleep hygiene education using the ASBQ to target maladaptive sleep behaviors might effectively enhance sleep indices in elite male cricket athletes [11].

This is the first reliability generalization meta-analysis study assessing the consistency and test-retest reliability of the ASBQ. The comprehensive search strategy and the evaluation of the methodological quality of the included studies, as well as the lack of publication bias are strengths of the present study. Nevertheless, some limitations should be acknowledged. First, an important limitation of the current reliability generalization meta-analysis is the small number of eligible studies meeting inclusion criteria during the specified time period (K = 5). Though meta-analysis is possible with as few as two combined studies, additional data points tend to yield more stable mean effect size estimates along with narrower confidence intervals. A contributing issue here was the substantial heterogeneity in observed Cronbach’s alpha values across the small set of available studies. Such variability reduces generalizability and indicates the pooled estimate should be interpreted cautiously. These reliability inconsistencies may stem from variations in methodology, samples, or other study parameters. However, with few included studies, parsing out sources of variability via moderator analysis remained difficult. Moving forward, expanding the evidence base through additional well-designed primary studies assessing the target instrument’s internal consistency would be beneficial. In particular, increasing overall sample size beyond the minimal benchmark of two studies is advisable when feasible, as findings from just six investigations with wide-ranging estimates provided insufficient precision for definitive conclusions regarding the true reliability level or factors influencing the degree of score consistency. Attempts at future meta-analytic updates may obtain more precise and consistent reliability figures as the number of published studies surpasses the tentative threshold of ten identified here.

Second, the included studies varied in terms of the athlete samples, sports, and level of competition. Future research could focus on conducting similar analyses within more homogenous subsets of athletes to ascertain the validity of these reliability estimates within specific populations. Third, the limited number of published studies warrants consideration. An update of this meta-analysis should be conducted after the publication of additional ASBQ validation studies. Last, the present meta-analysis exclusively examined Cronbach’s alpha and ICC as measures of reliability. Future meta-analyses should explore other psychometric properties, such as McDonald’s Omega, to provide a more comprehensive evaluation of the ASBQ’s overall effectiveness and applicability.

## Conclusion

In conclusion, this meta-analysis provides valuable insights into the reliability of the ASBQ. Despite its only acceptable internal consistency, its high test-retest reliability suggests that it can be a useful tool for assessing sleep behavior over time in athletes. Further research is needed to refine and optimize the ASBQ to ensure its applicability and validity in diverse athletic populations.

## Data Availability

Data is available in Table 1.

## Declarations

### Statement from authors

All authors have seen and approved the manuscript.
